# A comparison of commercial light-emitting diode baited suction traps for surveillance of *Culicoides* in northern Europe

**DOI:** 10.1186/s13071-015-0846-x

**Published:** 2015-04-22

**Authors:** Andrew Hope, Simon Gubbins, Christopher Sanders, Eric Denison, James Barber, Francesca Stubbins, Matthew Baylis, Simon Carpenter

**Affiliations:** Vector-borne Viral Disease Programme, The Pirbright Institute, Pirbright, Surrey United Kingdom; Liverpool University Climate and Infectious Diseases of Animals (Lucinda) Group, University of Liverpool, Neston, Cheshire United Kingdom; Health Protection Research Unit in Emerging and Zoonotic Infections, University of Liverpool, Liverpool, United Kingdom

**Keywords:** Ceratopogonidae, Bluetongue virus, Schmallenberg virus, Orbivirus, Surveillance, Diptera, *Culicoides*

## Abstract

**Background:**

The response of *Culicoides* biting midges (Diptera: Ceratopogonidae) to artificial light sources has led to the use of light-suction traps in surveillance programmes. Recent integration of light emitting diodes (LED) in traps improves flexibility in trapping through reduced power requirements and also allows the wavelength of light used for trapping to be customized. This study investigates the responses of *Culicoides* to LED light-suction traps emitting different wavelengths of light to make recommendations for use in surveillance.

**Methods:**

The abundance and diversity of *Culicoides* collected using commercially available traps fitted with Light Emitting Diode (LED) platforms emitting ultraviolet (UV) (390 nm wavelength), blue (430 nm), green (570 nm), yellow (590 nm), red (660 nm) or white light (425 nm – 750 nm with peaks at 450 nm and 580 nm) were compared. A Centre for Disease Control (CDC) UV light-suction trap was also included within the experimental design which was fitted with a 4 watt UV tube (320-420 nm). Generalised linear models with negative binomial error structure and log-link function were used to compare trap abundance according to LED colour, meteorological conditions and seasonality.

**Results:**

The experiment was conducted over 49 nights with 42,766 *Culicoides* caught in 329 collections. *Culicoides obsoletus* Meigen and *Culicoides scoticus* Downes and Kettle responded indiscriminately to all wavelengths of LED used with the exception of red which was significantly less attractive. In contrast, *Culicoides dewulfi* Goetghebuer and *Culicoides pulicaris* Linnaeus were found in significantly greater numbers in the green LED trap than in the UV LED trap. The LED traps collected significantly fewer *Culicoides* than the standard CDC UV light-suction trap.

**Conclusions:**

Catches of *Culicoides* were reduced in LED traps when compared to the standard CDC UV trap, however, their reduced power requirement and small size fulfils a requirement for trapping in logistically challenging areas or where many traps are deployed at a single site. Future work should combine light wavelengths to improve trapping sensitivity and potentially enable direct comparisons with collections from hosts, although this may ultimately require different forms of baits to be developed.

**Electronic supplementary material:**

The online version of this article (doi:10.1186/s13071-015-0846-x) contains supplementary material, which is available to authorized users.

## Background

*Culicoides* biting midges (Diptera: Ceratopogonidae) are responsible for the transmission of economically important arboviruses including bluetongue virus (BTV), African horse sickness virus (AHSV) and the newly emerged Schmallenberg virus (SBV) [1,2]. An accurate assessment of the phenology, abundance and distribution of *Culicoides* is important for understanding the potential transmission of BTV, AHSV and SBV and in monitoring disease outbreaks [3]. As a result, large-scale monitoring of *Culicoides* populations is carried out in many European countries and these are entirely reliant on overnight collections from ultraviolet light-suction (UVLS) traps of various designs [4]. In addition, UVLS traps have also been used in a vast array of behavioural studies across the region at a variety of spatial scales (e.g. between farm: [5]; within farms: [6-8]), largely in response to the incursions of BTV and SBV into Europe. While these traps are known to be unrepresentative of host biting rates on livestock in the region [9-11], they provide a rapid and logistically straightforward means of sampling *Culicoides* populations.

In the early stages of systematic *Culicoides* surveillance in southern Europe there was a divergence in the design of UVLS traps used in monitoring. In Italy [12], France [13], Switzerland [14,15] and many other countries, the Onderstepoort Veterinary Institute (OVI) trap design was used. This is a robust trap design weighing over 4 kg without a battery that is baited with an 8 watt 30 cm UV tube and uses an 11 cm diameter fan for suction. The OVI UVLS traps has been demonstrated to catch vast numbers of some species (e.g. over one million *Culicoides imicola* Kieffer have been trapped in a single night in South Africa [16]) and, anecdotally, has a strong reliability record in the case of the mains electricity powered model. There are issues, however, both in the commercial production of the OVI light-suction trap for what is a very limited market (these traps are used solely for *Culicoides* surveillance) and in the use of multiple traps for behavioural studies in the field due to its bulk and mains power requirements.

The Centre for Disease Control (CDC) UVLS trap (design 1212 and the similar 912) is also used for monitoring *Culicoides* populations in large-scale surveillance schemes, particularly in Spain [17]. This trap is baited with a 4 W 13.5 cm UV tube and uses an 8 cm fan for suction resulting in an overall power consumption of approximately 0.86 amps/hour (equating to a single night of trapping using a 6 volt/10 amp hours battery). The CDC UVLS (1212) is substantially lighter in weight than the OVI UVLS, weighing approximately 750 g excluding battery and hence more suitable for deployment in behavioural studies on a local scale. This trap is often used for large-scale monitoring of mosquitoes and hence is commercially sustainable. The CDC UVLS (1212) trap has, not surprisingly, been demonstrated to catch fewer *Culicoides* than the OVI UVLS trap, presumably due to its less powerful light bait and suction [18,19].

The development of low-cost light emitting diodes (LEDs) as a source of light has provided an opportunity to address some of the deficiencies of conventional light baits [20]. A major advantage in using LEDs is the reduced power consumption of these light sources when compared with standard incandescent and UV light sources. The wide range of applications for these traps has also enabled commercial development, although use remains limited at present in comparison to conventional UVLS traps. At present the Bioquip model 2770 represents the extreme of increased portability and reduced power consumption, based on an original design trialled on phlebotomine sand flies [21]. This trap weighs <500 g without a battery and operates with 8 LED elements to provide 360° coverage, but only in a horizontal plane, with a viewing angle of 45°. Power draw, even in comparison to the CDC UVLS (1212) trap, is minimal with a consumption of 0.35 amps/hour, (allowing three nights of trapping to be conducted with a single 6 Volt/10 amp hours battery charge). The unit can also be customised with LEDs of specific wavelength allowing certain species with preferences for a specific wavelength to be targeted.

Field studies have already used LEDs to assess the response of *Culicoides* to different wavelengths of light. LED-baited LS traps are currently used for surveillance of *Culicoides* in Australia, prompted by the appearance of Akabane virus in areas lacking the principal vector *C. brevitarsis* Kieffer in incandescent light-baited trap catches [22]. This observation led to a hypothesis that *C. brevitarsis* was under-represented by this trapping method and a green LED baited trap was subsequently found to collect significantly greater numbers, in addition to a greater diversity of *Culicoides* species than the standard incandescent control or blue- or UV-LED baited traps [23]. As a result of these findings, and the fact that LED-based traps consume less power making trapping logistics more straightforward, the Australian National Arbovirus Monitoring Program now employs green LED traps for monitoring *C. brevitarsis* in low density areas [24].

In this trial we examine the potential to use commercially available and highly portable LED-baited light-suction traps to collect *Culicoides* on a farm in the UK. Different light wavelength modules are used to investigate their impact on species diversity and abundance, and compared with a standard CDC UVLS. Conclusions regarding the possible role for LED-baited traps are then made along with potential for further improvement as tools to study *Culicoides* behaviour.

## Methods

### Study site

The trial was conducted from May to September 2011 at a farm in Surrey, south east England (51°20′09.60″N, 0°33′55.87″W). The site comprised a large field (140 m × 120 m) subdivided into smaller grazing enclosures that in total accommodated four horses and two pigs. Two sides of the site were surrounded by deciduous woodland and two sides bordered further grazing land used for horses.

### Collection methods

**Figure 1:**
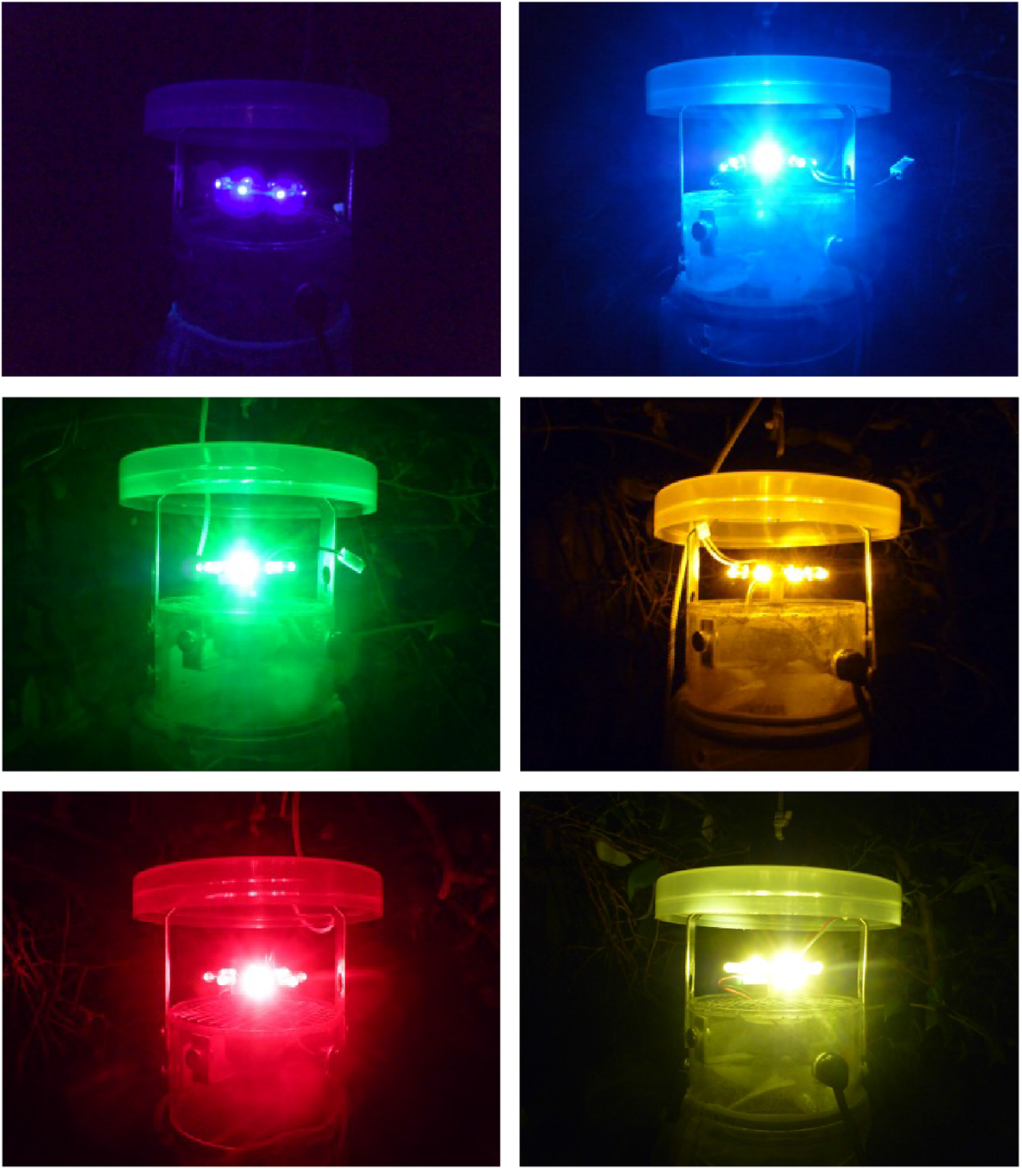
Light emitting diode baits used during investigation of differential attraction of *Culicoides* to commercially produced light-suction traps. Colours used were: UV (390 nm), Blue (430 nm), Green (570 nm), Yellow (590 nm), Red (660 nm) and White (427-750 nm).

*Culicoides* were collected using LS traps (Model 2770, Bioquip Inc., USA) fitted with LED platforms consisting of 8 individual LEDs emitting at specific wavelengths [20]. Six different colours of LED were used in the traps (Figure 1): ultraviolet (390 nm); Blue (430 nm); Green (570 nm); Yellow (590 nm); Red (660 nm) and White (425 nm – 750 nm with peaks at 450 nm and 580 nm). An additional standard CDC LS trap (Model 912, J. W. Hock, USA) fitted with a 4 watt UV tube (320–420 nm) was used as a positive control. Traps were hung at a height of 1.5 m, with an inter-trap distance of at least 50 m. *Culicoides* attracted to the light traps were collected into beakers containing 200 ml of water and a drop of detergent. Following collection samples were drained through a sieve and transferred to 70% ethanol for storage.

Traps using 6 volt batteries were operated from late afternoon until the following morning in order to encompass the sunset and sunrise peaks in UK *Culicoides* activity [25]. On night 1 the trap treatments were randomly assigned to locations and on subsequent nights the treatments were rotated to the next location in a clockwise direction. After seven nights of trapping, all traps had collected at each of the seven locations and the treatments were again re-randomised to trap locations for the start of the next rotation. A total of seven rotations were completed giving 49 nights of data collection.

### Meteorological data

Meteorological data were collected throughout the sampling period using an automatic weather station (CR800 data logger, Campbell Scientific, UK). Data collected were: air temperature (°C); relative humidity (%); solar intensity (Wm^−2^); wind speed (ms^−1^) and wind direction (°). For data analysis, wind direction was transformed using the ArcTangent2 function in Excel as it is a circular variable and therefore wind direction at 0° and 360° represent the same direction.

### Sample identification

Trapped *Culicoides* were initially sorted using morphological characteristics according to physiological status into unpigmented, pigmented, gravid and blood-fed individuals. While *C. chiopterus* Meigen was identified by morphology (small size and pale markings on wings), other female members of the subgenus Avaritia (*C. obsoletus*, *C. scoticus* and *C. dewulfi*) were identified using a modified multiplex polymerase chain reaction (PCR) [26,27]. To extract DNA, *Culicoides* were removed from 70% ethanol storage and allowed to dry on paper towelling for 10 minutes, before being placed individually into 2 ml micro-collection tubes (Qiagen, UK). Ten microlitres of 2% proteinase-k (Bioline, UK) (made in solution with tris calcium acetate) was then added, along with 200 μl of 5% chelex (Bio-Rad, UK). Samples were homogenised in two cycles of two minutes at 25 Hz in a Tissuelyser (Qiagen, UK) and then incubated overnight at 37°C. Following incubation, 4 μl of each sample was removed and added to a PCR plate (Abgene, UK). Samples were subjected to an eight minute cycle at 99°C to de-activate the proteinase-k. PCR mastermix for each PCR plate consisted: 25 μl of 10 μM forward primer specific to each species (*C. obsoletus*: TGCAGGAGCTTCTGTAGATTTG; *C. scoticus*: ACCGGCATAACTTTTGATCG; *C. dewulfi*: ATACTAGGAGCGCCCGACAT) [26]; 25 μl DNAse free water; 100 μl of 10 μM universal reverse primer (CAGGTAAAATTAAAATATAAACTTCTGG) [27]; 7 μl MgCl_2_ solution (Bioline, UK) and 400 μl Biomix Red solution (Bioline, UK).

Each 4 μl sample of extracted DNA had 6 μl of mastermix added. In addition to the test samples, each plate also contained 3 positive controls, using DNA extracted from males of each species using spin column methods according to the manufacturer’s protocol (Qiagen, UK), and 3 negative controls, which consisted of 4 μl of DNAse free water. Samples were then placed in a thermocycler with the following profile: initial denaturing step at 94°C for 4 minutes; 32 cycles of 94°C for 30 seconds, 60°C for 30 seconds, 72°C for 1 minute; followed by a final extension step at 72°C for 5 minutes [27]. Polymerase chain reaction products were examined by electrophoresis using 2% agarose e-gels (Invitrogen, UK) and these were compared to a DNA ladder (Invitrogen, UK) for means of identification.

Due to the large number of *Culicoides* collected it was not possible to process all individuals to species level using the diagnostic PCR and a sub-sampling method was used. For each trap treatment, two nights were randomly selected from each seven nights rotation and all *C. obsoletus*/*C. scoticus*/*C.dewulfi* females within the trap catch were identified by PCR to species level. The PCR results were then combined and for each seven night rotation the proportions of each species were applied to the collections from the remaining five nights of the rotation. If collections failed to amplify then another night was randomly selected for analysis and failed samples were excluded from final estimates.

### Statistical analysis

Generalised linear models (GLMs) with negative binomial error structure and log-link function were constructed in R (version 2.15.2) to compare *Culicoides* collections according to the wavelengths of light used as bait. Initial GLMs included all meteorological variables: air temperature (°C); relative humidity (%); solar radiation (Wm^−2^); wind speed (ms^−1^); transformed wind direction and variation in wind direction (°). Linear and quadratic temporal trends were included to model the effect of seasonality on collections. Trap site was included as an explanatory variable to account for any variation between the seven trap locations. The construction of final models was preceded by stepwise deletion of non-significant (p > 0.05) variables, with the final model corresponding to the one where all terms were significant. Differences in mean trap catch size between the different traps were then assessed using Tukey’s all-pairs comparison test.

## Results

Sampling was conducted over 49 nights, giving a total of 329 successful collections, after the exclusion of 14 trap failures (4.1% of total catches: 12 due to motor failures on LED traps; 1 due to battery failure on CDC LS trap; 1 due to operator error). A total of 42,766 *Culicoides* were collected, the majority of which were females of *C. obsoletus*, *C. scoticus* and *C. dewulfi*, accounting for 84.3% of the trap collection (Table 1). Males of these species were far less commonly caught, constituting 3.2% of the total collection. Other species collected in order of abundance were *C. brunnicans* Edwards (4.8%); *C. pulicaris* (2.1%); *C. punctatus* Meigen (0.9%); and *C. impunctatus* Goetghebuer (0.8%). The remaining 3.9% of individuals included rarer species (*C. achrayi* Kettle and Lawson, *C. festivipennis* Kieffer*, C. pictipennis *Staeger,* C. nubeculosus Meigen and C. chiopterus*). The species diversity of *Culicoides* collected per trap according to light wavelength are presented in Table 1.

**Table 1:** ***Culicoides*** **biting midges collected during a comparative study of commercially available light emitting diode baited suction traps. Studies were conducted over a total of 49 days at a farm holding in the United Kingdom**

**Species**	**Total** ***Culicoides*** **collected (Mean ± SEM)**							
	**CDC (n = 48)**	**Ultraviolet (n = 47)**	**Blue (n = 46)**	**Green (n = 49)**	**Yellow (n = 48)**	**Red (n = 45)**	**White (n = 46)**	**Total (n = 329)**
*C. obsoletus/C. scoticus/C. dewulfi*	20,569 (429 ± 110)	3,077 (65.5 ± 18.6)	3,515 (76.4 ± 24.1)	3,965 (80.9 ± 17.3)	2,810 (58.5 ± 25.0)	122 (2.7 ± 0.6)	3,379 (73.5 ± 23.0)	**37,437**
*C. pulicaris*	389 (8.1 ± 2.8)	49 (1.0 ± 0.3)	119 (2.6 ± 0.9)	157 (3.2 ± 1.0)	69 (1.4 ± 0.5)	1 (0.02 ± 0.0)	100 (2.2 ± 0.7)	**884**
*C. punctatus*	210 (4.4 ± 1.7)	20 (0.4 ± 0.2)	55 (1.2 ± 0.9)	77 (1.6 ± 0.5)	31 (0.6 ± 0.3)	0	13 (0.3 ± 0.1)	**406**
*C. impunctatus*	93 (1.9 ± 0.8)	54 (1.1 ± 0.6)	91 (2.0 ± 1.2)	72 (1.5 ± 0.4)	16 (0.3 ± 0.1)	2 (0.04 ± 0.0)	16 (0.3 ± 0.1)	**344**
*C. brunnicans*	264 (12.6 ± 4.9)	103 (4.9 ± 1.9)	542 (25.8 ± 22.4)	744 (35.3 ± 27.3)	213 (10.1 ± 5.3)	19 (1.0 ± 0.4)	186 (10.3 ± 5.5)	**2,071**
Other Species	527	154	319	357	164	3	100	**1,624**
**Total**	**22,052**	**3,457**	**4,641**	**5,372**	**3,303**	**147**	**3,794**	**42,766**

A total of 9,918 female individuals identified morphologically as being one of either *C. obsoletus*, *C. scoticus* or *C. dewulfi* were subjected to molecular identification by multiplex PCR, of which 88.9% were successfully identified and 11.1% failed due to poor DNA extraction. Of the 8,853 individuals successfully identified, 5,862 (66.2%) were *C. obsoletus*, 2,789 (31.5%) were *C. scoticus* and 202 (2.3%) were *C. dewulfi*. The sub-sampling results were then used to calculate the total numbers for these species per physiological status as shown in Table 2; samples that failed to amplify in the PCR are not included and six males belonging to the subgenus Avaritia are also excluded as these were damaged and could not be identified to species level. Numbers of *C. obsoletus* and *C. scoticus* females collected were sufficient for three models for each species to be constructed for analysis: total females (includes all physiological states); unpigmented females and pigmented females. For *C. dewulfi*, *C. pulicaris* and *C. brunnicans*, analyses were restricted to single models for total females of all physiological states due to the smaller number of individuals collected. Full details of the final models for each species are presented in Additional file 1: Tables S1-S6.

**Table 2:** **Estimated abundances of** ***C. obsoletus*** **,** ***C. scoticus*** **and** ***C. dewulfi*** **based on multiplex polymerase chain reaction identification of subsamples**

**Species**	**Physiological status**	**CDC**	**UV**	**Blue**	**Green**	**Yellow**	**Red**	**White**	**Total**
*C. obsoletus*	Unpigmented	7,336	1,259	1,351	1,628	979	24	1,458	14,035
	Pigmented	2,545	604	752	814	529	21	537	5,802
	Blood-fed	92	9	44	72	46	0	96	359
	Gravid	219	42	153	189	123	1	27	754
	Male	384	62	97	209	37	8	88	885
	Total	10,576	1,976	2,397	2,912	1,714	54	2,206	21,835
*C. scoticus*	Unpigmented	4,826	489	301	316	552	28	381	6,893
	Pigmented	4,405	425	307	441	310	21	252	6,161
	Blood-fed	3	1	7	17	4	0	12	44
	Gravid	96	6	28	3	9	1	30	173
	Male	264	24	41	56	37	3	24	449
	Total	9,594	945	684	833	912	53	699	13,720
*C. dewulfi*	Unpigmented	147	14	45	19	33	0	40	298
	Pigmented	92	7	70	36	22	2	37	266
	Blood-fed	2	0	37	3	0	0	0	42
	Gravid	86	19	13	38	1	0	1	158
	Male	17	6	2	10	3	2	6	46
	Total	344	46	167	106	59	4	84	810
**Total**		**20,514**	**2,967**	**3,248**	**3,851**	**2,685**	**111**	**2,989**	**36,365**

After allowing for the effects of meteorological variables, trap location and temporal trends the CDC trap collected significantly more *C. obsoletus* and *C. scoticus* of all physiological types than any of the other traps (p < 0.01) (S.2. and S.4.). The one exception was for the collection of pigmented *C. obsoletus* using the green-wavelength light-suction trap where there was no statistical difference with the CDC trap, although this result was marginal (p = 0.057). Furthermore, the red trap collected significantly fewer individuals than all other traps (p < 0.001).

CDC UVLS trap collections of *C. dewulfi* were not significantly different to the blue and green LED LS traps (p > 0.05) (Additional file 1: Table S6). Amongst the LED light-suction traps there were no significant differences between the blue and green-baited LS traps, but both collected significantly greater numbers than the UV, yellow and red LED LS traps (p < 0.05). The red LED baited LS trap collected significantly fewer individuals than all other LS traps (p < 0.05).

Analysis of *C. pulicaris* collections showed a similar pattern to *C. dewulfi* with the CDC UVLS trap collections not being significantly greater in abundance than the blue and green LED baited LS trap collections (p > 0.05) and no difference was observed between the blue and green-baited LED LS traps (Additional file 1: Table S6). The green-baited LED LS trap also collected significantly greater numbers than the UV, yellow and red LED baited LS traps (p < 0.01) and the red-baited LED LS trap consistently collected fewer *C. pulicaris* than all other LS traps. In the *C. brunnicans* analysis there was also no difference in collections made using the CDC UVLS trap compared to the green and blue LED baited LS traps and there was also no difference to the white LED baited LS trap (p < 0.05) (see Additional file 1: Tables S5 and S6.). Among the LED-baited LS traps no differences were observed between the blue, green and UV-baited LED LS traps, but the red LED LS trap collected significantly fewer *C. brunnicans* than all other traps with the exception of the UV LED LS trap where no significant difference was found. This species was restricted to the early part of the season in contrast to the other abundant species that were present throughout the trial (Figure 1).

**Figure 2:**
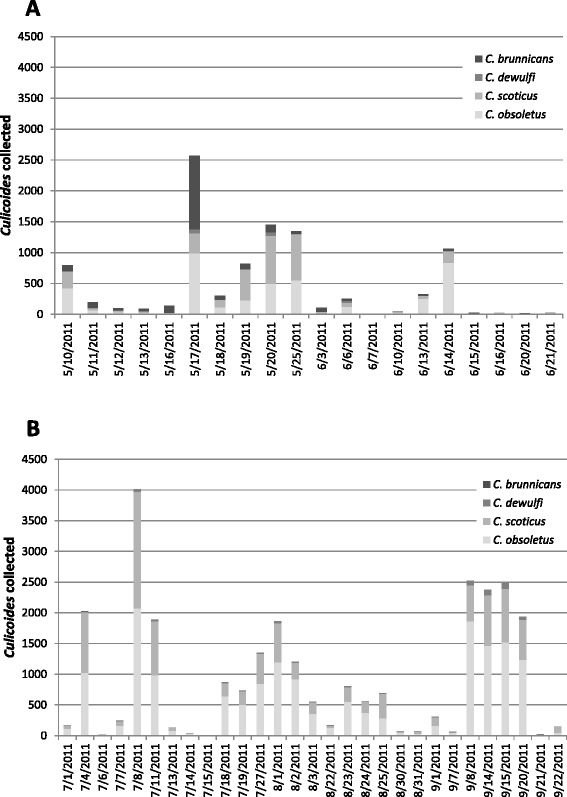
Seasonal occurrence of common *Culicoides* species from **(A)** May to June and **(B)** July to September. Collections are pooled across suction trap catches using light emitting diodes and a conventional ultraviolet light baited trap. Abundance for *C. obsoletus*, *C. scoticus* and *C. dewulfi* is calculated from a subsample of specimens identified to species level using a multiplex polymerase chain reaction assay while *C. brunnicans* were identified directly using their morphology.

Collections of all species made at trap location 1 were typically higher than at other locations though the differences were not always statistically significant (see Additional file 1: Tables S1, S3 and S5). Where statistically significant the effects of meteorological variables were consistent across species (see Additional file 1: Tables S1, S3 and S5). Higher catches were associated with higher temperatures, higher humidity, lower solar radiation and lower wind-speeds. Seasonal variation in populations was marked, with *C. brunnicans* activity restricted to the beginning of the adult season, while other species were present throughout the sampling period (Figure 2).

## Discussion

This is the first study to assess the performance of LED-baited LS traps in direct comparison to a standard surveillance tool for European *Culicoides* species. Throughout the study, the CDC UVLS trap (912) consistently outperformed the LED LS traps in the abundance of all *Culicoides* species collected, with the exception of *C. brunnicans*. Despite this, numbers collected by the LED LS traps were still substantial, with maximum single night catches exceeding 500 individuals for blue, green and white LED baits. Trap reliability, while poorer than that of the bulkier traps, was manageable across the field season and the reduced requirement for battery charging made carrying out the trapping substantially more straightforward. LED LS traps may therefore play a role in studies requiring intensive trapping at a single site where the daily recharging of batteries is prohibitively time consuming. A second potential role the LED LS traps could fulfil is in sampling of *Culicoides* populations prior to the establishment of major surveillance efforts, particularly in regions with limited, or intermittent, supply of mains electricity. Here, their portability would be of great use in reducing luggage weight and reduce power requirements which may enable sampling missions to be extended in the field.

Across the wavelengths of light tested, the most consistent pattern of *Culicoides* response was a poor attraction to the red (660 nm) LED LS trap. Excluding the red LED LS trap, *C. obsoletus* and *C. scoticus* appeared to exhibit an indiscriminate response to the different LED LS traps used. In contrast, while *C. dewulfi* was collected in fewer numbers than *C. obsoletus* and *C. scoticus*, the blue and green LED LS traps collected significantly more than the UV LED LS trap. This demonstrates that *C. dewulfi* shows differential attraction to traps emitting different wavelengths and that the use of UV light baited traps may risk under-estimating the population of this species. Very similar results were also found in the collection of *C. pulicaris* and *C. brunnicans*. All of these species demonstrated parallels to *C. brevitarsis* in Australia which was previously found to respond to green LED baits [23]. While the experimental design used a clockwise rotation of traps rather than a more ideal randomised allocation of placement it is assumed that this did not greatly influence results due to the high degree of replication (7 full rotations with random initial allocation of sites).

While the relative influence of each northern European species of *Culicoides* in transmission of BTV and SBV remains uncertain [2,28], it is unlikely that these differences are sufficient to warrant alteration of light wavelengths used for standard surveillance. As only commercially available traps were used in the present study no attempt was made to standardise for the intensity of light emitted by each trap. Further investigation using LEDs with a uniform light intensity would reveal whether or not the green wavelength is inherently superior to UV for trapping *C. dewulfi*, *C. pulicaris* and *C. brunnicans*. A separate laboratory study to investigate spectral sensitivity through electroretinograms as conducted with sandflies may also yield useful information on the response of *Culicoides* to different light wavelengths [29]. An additional option for increasing the sensitivity of catches would be to combine LEDs of different wavelengths within a single unit. This could have the advantage of increased detection of green/blue sensitive species, which at present may be under-represented in UV LS trap catches.

The study site chosen for the trial contained large populations of most of the common livestock-associated species of *Culicoides* in the UK, confirmed through the use of the control CDC light-suction trap. These collections were dominated by *C. obsoletus* and *C. scoticus*, which are ubiquitous across Europe, with a lesser abundance of *C. dewulfi*, *C. brunnicans*, *C. pulicaris, C. punctatus* and *C. impunctatus*, all of which have been recorded in previous trials carried out locally to this region [30]. It was notable, however, that cattle-dung breeding species (namely *C. dewulfi* and *C. chiopterus*) were under-represented as a proportion of total catch when compared to farm studies conducted elsewhere in northern Europe [31]. This may have been due to the close relationship between these species and cattle [32], which were not directly present at the site during the trial (although they were occasionally grazed in adjacent fields to the study area). As expected, flight behaviour of *Culicoides* was heavily influenced by meteorological variables making it essential to include these data in any analysis of field collections of these species [9,33]. The models generated are broadly in agreement with previous *Culicoides* studies in showing that temperature and humidity have a positive impact on trap collections whereas wind speed has a negative impact.

A challenge for interpretation of LS trap catches of *Culicoides* is in assessing the abundance of individuals collected against those biting hosts. There is clear evidence that this relationship is both complex and varies considerably from study to study [9-11]. Comparative studies with other and combined LED wavelengths may yield improvements in this comparability; however, the intrinsic limitation of light based sampling methods may require alternative methods to be used, not least the fact that light traps are effective for only part of the adult active period of many *Culicoides* species. These methods could include the use of semiochemical-based approaches as already trialled in northern Europe that mimic host location cues, although these remain at a relatively early stage of development [34].

## Conclusions

The abundance of *Culicoides* collected was reduced in the LED LS traps when compared to the standard CDC UV LS trap, however, their reduced power requirement and small size fulfils a requirement for trapping in logistically challenging areas or where many traps are deployed at a single site. Future work should consider combining light wavelengths which may improve trapping sensitivity and direct comparisons with collections from hosts to examine comparability with biting rates.

## Additional file

Additional file 1:
**Table S1.** Regression coefficients for final models to describe collections of *C. obsoletus.* **Table S2.** Multiple Tukey’s all-pair comparisons of catch size between trap types for *C. obsoletus* females. **Table S3.** Regression coefficients for final models to describe collections of *C. scoticus.* **Table S4.** Multiple Tukey’s all-pair comparisons of catch size between trap types for *C. scoticus* females. **Table S5.** Regression coefficients for final models of females of *C. dewulfi*, *C. pulicaris* and *C. brunnicans.* **Table S6.** Multiple Tukey’s all-pair comparisons of catch size between trap types for *C. dewulfi*, *C. pulicaris* and *C. brunnicans* females.
