# Supplementary material for: A comparison of commercial light-emitting diode baited suction traps for surveillance of Culicoides in northern Europe
Source: Parasit Vectors. 2015 Apr 22;8:239. doi: 10.1186/s13071-015-0846-x (PMC4415440; doi:10.1186/s13071-015-0846-x)
Supplement: Additional file 1: — Table S1. Regression coefficients for final models to describe collections of C. obsoletus. Table S2. Multiple Tukey’s all-pair comparisons of catch size between trap types for C. obsoletus females. Table S3. Regression coefficients for final models to describe collections of C. scoticus. Table S4. Multiple Tukey’s all-pair comparisons of catch size between trap types for C. scoticus females. Table S5. Regression coefficients for final models of females of C. dewulfi, C. pulicaris and C. brunnicans. Table S6. Multiple Tukey’s all-pair comparisons of catch size between trap types for C. dewulfi, C. pulicaris and C. brunnicans females. [file 13071_2015_846_MOESM1_ESM.docx]

##### S.1. Regression coefficients for final models to describe collections of *C. obsoletus* (*=p<0.05, **=p<0.01, ***=p<0.001)

|  | *C. obsoletus* Total Females | | *C. obsoletus* Unpigmented | | *C. obsoletus* Pigmented | |
| --- | --- | --- | --- | --- | --- | --- |
| Parameter | Estimate | 95% C.I. | Estimate | 95% C.I. | Estimate | 95% C.I. |
| Intercept | -5.804*** | -8.483; -3.145 | -6.764*** | -9.558; -4.034 | -7.365*** | -10.280; -4.465 |
| Temporal Trend |  |  |  |  |  |  |
| Linear | -0.032*** | -0.048; -0.015 | NS | - | -0.046*** | -0.065; -0.027 |
| Quadratic | 0.0002*** | 0.00009; 0.0003 | NS | - | 0.0002*** | 0.0001; 0.003 |
| Trap |  |  |  |  |  |  |
| CDC | 1.657*** | 1.031; 2.279 | 1.669*** | 1.012; 2.323 | 1.571*** | 0.869; 2.243 |
| Blue | Baseline | Baseline | Baseline | Baseline | Baseline | Baseline |
| Green | 0.612 | -0.028; 1.245 | 0.613 | -0.070; 1.297 | 0.634 | -0.054; 1.323 |
| Red | -3.272*** | -4.000; -2.545 | -3.641*** | -4.481; -2.811 | -2.941*** | -3.804; -2.090 |
| UV | 0.203 | -0.444; 0.850 | 0.126 | -0.549; 0.802 | 0.262 | -0.433; 0.961 |
| White | 0.555 | -0.581; 0.691 | 0.122 | -0.565; 0.809 | 0.087 | -0.600; 0.776 |
| Yellow | -0.172 | -0.804; 0.459 | -0.202 | -0.892; 0.486 | 0.029 | -0.660; 0.719 |
| Trap Location |  |  |  |  |  |  |
| Position 1 | Baseline | Baseline | Baseline | Baseline | Baseline | Baseline |
| Position 2 | -0.365*** | -0.995; 0.264 | -0.280 | -0.953; 0.393 | -0.550 | -1.232; 0.131 |
| Position 3 | -1.172*** | -1.816; -0.528 | -0.915** | -1.582; -0.247 | -1.400*** | -2.102; 0.700 |
| Position 4 | -2.288 | -2.968; -1.606 | -2.674*** | -3.402; -1.939 | -2.374*** | -3.132; -1.619 |
| Position 5 | -0.522 | -1.170; -0.121 | -0.563 | -1.246; 0.116 | -0.731* | -1.442; -0.028 |
| Position 6 | -0.590 | -1.236; 0.054 | -0.587 | -1.266; 0.089 | -0.879** | -1.596; -0.166 |
| Position 7 | -2.685*** | -3.349; -2.017 | -2.687*** | -3.404; -1.964 | -2.819*** | -3.564; -2.074 |
| Temperature | 0.327*** | 0.227; 0.428 | 0.264*** | 0.170; -0.363 | 0.371*** | 0.257; 0.489 |
| Humidity | 0.063*** | 0.041; 0.086 | 0.060*** | 0.045; 0.091 | 0.068*** | 0.045; 0.092 |
| Solar Radiation | NS | - | -11.922** | -19.479; -3.756 | NS | - |
| Wind Speed | -0.481*** | -0.803; -0.156 | -0.344* | -0.672; -0.012 | -0.720*** | -1.087; -0.351 |
| Variation Wind Direction | 0.015*** | 0.003; 0.026 | 0.017** | 0.005; 0.028 | 0.0179*** | 0.005; 0.029 |

**S.2. Multiple Tukey’s all-pair comparisons of catch size between trap types for *C. obsoletus* females, estimates are for treatments on the top row relative to treatments in the left column (*=p<0.05, **=p<0.01, ***=p<0.001)**

| Trap | CDC | UV | Blue | Green | Yellow | Red |
| --- | --- | --- | --- | --- | --- | --- |
| UV | 1.454*** | - |  |  |  |  |
| Blue | 1.656*** | 0.202 | - |  |  |  |
| Green | 1.045** | 0.409 | -0.612 | - |  |  |
| Yellow | 1.828*** | 0.374 | 0.172 | 0.784 | - |  |
| Red | 4.929*** | 3.475*** | 3.272*** | 3.884*** | 3.100*** | - |
| White | 1.601*** | 0.147 | -0.055 | 0.556 | -0.227 | -3.327*** |

Total *C. obsoletus* females

| Trap | CDC | UV | Blue | Green | Yellow | Red |
| --- | --- | --- | --- | --- | --- | --- |
| UV | 1.543*** | - |  |  |  |  |
| Blue | 1.669*** | 0.126 | - |  |  |  |
| Green | 1.055* | -0.487 | -0.613 | - |  |  |
| Yellow | 1.872*** | 0.329 | 0.202 | 0.816 | - |  |
| Red | 5.310*** | 3.767*** | 3.641*** | 4.254*** | 3.438*** | - |
| White | 1.547*** | 0.004 | -0.122 | 0.491 | -0.324 | -3.763*** |

Unpigmented *C. obsoletus*

| Trap | CDC | UV | Blue | Green | Yellow | Red |
| --- | --- | --- | --- | --- | --- | --- |
| UV | 1.307** | - |  |  |  |  |
| Blue | 1.570*** | 0.262 | - |  |  |  |
| Green | 0.936 | -0.371 | -0.633 | - |  |  |
| Yellow | 1.541*** | 0.233 | -0.029 | 0.604 | - |  |
| Red | 4.511*** | 3.203*** | 2.941*** | 3.574*** | 2.970*** | - |
| White | 1.483*** | 0.175 | -0.087 | 0.546 | -0.058 | -3.028*** |

Pigmented *C. obsoletus*

##### S.3. Regression coefficients for final models to describe collections of *C. scoticus* (*=p<0.05, **=p<0.01, ***=p<0.001)

|  | *C. scoticus* Total Females | | *C. scoticus* Unpigmented | | *C. scoticus* Pigmented | |
| --- | --- | --- | --- | --- | --- | --- |
| Parameter | Estimate | 95% C.I. | Estimate | 95% C.I. | Estimate | 95% C.I. |
| Intercept | -5.683*** | -8.661; -2.721 | -2.904* | -5.165; -6.040 | -7.556*** | -10.816; -4.340 |
| Temporal Trend |  |  |  |  |  |  |
| Linear | -0.032*** | -0.051; -0.012 | NS | - | -0.053*** | -0.073; -0.033 |
| Quadratic | 0.0002*** | 0.00008; 0.0003 | 0.00004* | 0.000005; 0.00007 | 0.0003*** | 0.0001; 0.0004 |
| Trap |  |  |  |  |  |  |
| CDC | 2.988*** | 2.314; 3.662 | 3.035*** | 2.328; 3.739 | 2.993*** | 2.288; 3.695 |
| Blue | Baseline | Baseline | Baseline | Baseline | Baseline | Baseline |
| Green | 0.789* | 0.089; 1.486 | 0.688 | -0.046; 1.422 | 0.821* | 0.095; 1.546 |
| Red | -2.079*** | -2.855; -1.305 | -2.205*** | -3.078; -1.338 | -2.224*** | -3.112; -1.349 |
| UV | 0.928** | 0.2403; 1.619 | 0.813* | 0.088; 1.538 | 0.837* | 0.113; 1.565 |
| White | 0.512 | -0.192; 1.216 | 0.634 | -0.114; 1.381 | 0.339 | -0.400; 1.080 |
| Yellow | 0.314 | 0.378; 1.007 | 0.343 | -0.395; 1.080 | 0.168 | -0.564; 0.901 |
| Trap Location |  |  |  |  |  |  |
| Position 1 | Baseline | Baseline | Baseline | Baseline | Baseline | Baseline |
| Position 2 | -0.448 | -1.150; 0.255 | -0.148 | -0.873; 0.579 | -0.609 | -1.331; 0.113 |
| Position 3 | -1.162*** | -1.879; -0.451 | -0.993** | -1.707; -0.279 | -1.270*** | -2.016; -0.529 |
| Position 4 | -2.150*** | -2.903; -1.401 | -0.240*** | -3.209; -1.593 | -2.171*** | -2.962; -1.384 |
| Position 5 | -0.579 | -1.281; 0.116 | -0.475 | -1.198; 0.246 | -0.548 | -1.268; 0.164 |
| Position 6 | -0.868** | -1.557; -0.180 | -0.704* | -1.413; 0.006 | -0.907** | -1.626; -0.191 |
| Position 7 | -2.943*** | -3.659; -2.226 | -2.892*** | -3.663; -2.116 | -2.807*** | -3.573; -2.041 |
| Temperature | 0.212*** | 0.101; 0.324 | NS | - | 0.300*** | 0.183; 0.419 |
| Humidity | 0.063*** | 0.039; 0.087 | 0.046*** | 0.023; 0.068 | 0.067*** | 0.042; 0.093 |
| Solar Radiation | -9.020* | -17.156; -3.035 | -16.38*** | -24.910; -7.228 | NS | - |
| Wind Speed | -0.462** | -0.805; -0.115 | NS | - | -0.617*** | -0.993; -0.240 |
| Variation Wind Direction | 0.018*** | 0.0069; 0.0265 | 0.013** | -0.002; 0.023 | 0.021*** | 0.009; 0.033 |

**S.4. Multiple Tukey’s all-pair comparisons of catch size between trap types for *C. scoticus* females, estimates are for treatments on the top row relative to treatments in the left column (*=p<0.05, **=p<0.01, ***=p<0.001)**

| Trap | CDC | UV | Blue | Green | Yellow | Red |
| --- | --- | --- | --- | --- | --- | --- |
| UV | 2.059*** | - |  |  |  |  |
| Blue | 2.988*** | 0.928 | - |  |  |  |
| Green | 2.199*** | 0.140 | -0.788 | - |  |  |
| Yellow | 2.673*** | 0.614 | -0.314 | 0.474 | - |  |
| Red | 5.067*** | 3.008*** | 2.079*** | 2.868*** | 2.393*** | - |
| White | 2.476*** | 0.417 | -0.511 | 0.277 | -0.197 | -2.590*** |

Total *C. scoticus* females

| Trap | CDC | UV | Blue | Green | Yellow | Red |
| --- | --- | --- | --- | --- | --- | --- |
| UV | 2.222*** | - |  |  |  |  |
| Blue | 3.035*** | 0.813 | - |  |  |  |
| Green | 2.346*** | 0.124 | -0.688 | - |  |  |
| Yellow | 2.691*** | 0.469 | -0.343 | 0.345 | - |  |
| Red | 5.240*** | 3.018*** | 2.204*** | 2.893*** | 2.548*** | - |
| White | 2.401*** | 0.179 | -0.633 | 0.054 | -0.290 | -2.838*** |

Unpigmented *C. scoticus*

| Trap | CDC | UV | Blue | Green | Yellow | Red |
| --- | --- | --- | --- | --- | --- | --- |
| UV | 2.156*** | - |  |  |  |  |
| Blue | 3.035*** | 0.837 | - |  |  |  |
| Green | 2.171*** | 0.154 | -0.821 | - |  |  |
| Yellow | 2.825*** | 0.669 | -0.168 | 0.653 | - |  |
| Red | 5.217*** | 3.061*** | 2.224*** | 3.045*** | 2.392*** | - |
| White | 2.654*** | 0.498 | -0.339 | 0.482 | -0.170 | -2.563*** |

Pigmented *C. scoticus*

##### S.5. Regression coefficients for final models of females of *C. dewulfi*, *C. pulicaris* and *C. brunnicans* (*=p<0.05, **=p<0.01, ***=p<0.001)

|  | *C. dewulfi* Total Females | | *C. pulicaris* Total Females | | *C. brunnicans* Total Females | |
| --- | --- | --- | --- | --- | --- | --- |
| Parameter | Estimate | 95% C.I. | Estimate | 95% C.I. | Estimate | 95% C.I. |
| Intercept | -11.132*** | -15.570; -7.277 | -2.617 | -5.719; 0.477 | -14.799*** | -20.174; -9.591 |
| Temporal Trend |  |  |  |  |  |  |
| Linear | -0.083*** | -0.112; -0.056 | 0.008** | 0.002; 0.015 | NS | - |
| Quadratic | 0.0005*** | 0.0003; 0.0007 | NS | - | -0.003** | -0.004; -0.002 |
| Trap |  |  |  |  |  |  |
| CDC | 1.033** | 0.220; 1.842 | 0.957** | 0.255; 1.659 | 1.345*** | 0.297; 2.403 |
| Blue | Baseline | Baseline | Baseline | Baseline | Baseline | Baseline |
| Green | 0.415 | -0.396; 1.222 | 0.506 | -0.236; 1.260 | 1.000* | -0.031; 2.033 |
| Red | -4.549*** | -7.003; -2.817 | -4.743*** | -7.697; -3.002 | -1.843** | -3.062; -0.614 |
| UV | -1.423*** | -2.397; -0.498 | -0.788* | -1.572; -0.005 | -0.324 | -1.368; 0.738 |
| White | -0.157 | -1.019; 0.701 | -0.051 | -0.791; 0.689 | 0.291 | -0.723; 1.310 |
| Yellow | -1.434*** | -2.392; -0.498 | -0.793* | -1.565; -0.028 | -0.087 | -1.096; 0.919 |
| Trap Location |  |  |  |  |  |  |
| Position 1 | Baseline | Baseline | Baseline | Baseline | Baseline | Baseline |
| Position 2 | -1.019* | -1.849; -0.192 | -0.867* | -1.566; -0.164 | 0.914 | -0.021; 1.856 |
| Position 3 | -2.033*** | -2.932; -1.142 | -1.088** | -1.812; -0.363 | 1.017* | 0.032; 2.011 |
| Position 4 | -2.958*** | -4.033; -1.911 | -2.958*** | -3.886; -2.063 | -1.358* | -2.431; -0.278 |
| Position 5 | -0.574 | -1.381; 0.226 | -1.045** | -1.732; -0.361 | 0.067 | -0.948; 1.104 |
| Position 6 | -1.268** | -2.133; -0.411 | -1.375*** | -2.085; -0.665 | -0.101 | -1.147; 0.949 |
| Position 7 | -2.980*** | -4.037; -1.955 | -3.940*** | -4.989; -2.961 | -1.892*** | -3.134; -0.644 |
| Temperature | 0.442*** | 0.289; 0.602 | 0.111* | 0.005; 0.215 | 1.159*** | 0.834; 1.515 |
| Humidity | 0.085*** | 0.056; 0.122 | 0.029* | 0.002; 0.057 | 0.047* | 0.006; 0.087 |
| Solar Radiation | NS | - | -12.651* | -21.910; -3.101 | NS | - |
| Wind Speed | -0.627** | -1.112; -0.145 | NS | - | -1.453*** | -2.330; -0.636 |
| Variation Wind Direction | 0.020** | -0.003; 0.037 | 0.002* | -0.0002; 0.004 | -0.003* | -0.006; -0.00003 |

**S.6. Multiple Tukey’s all-pair comparisons of catch size between trap types for *C. dewulfi*, *C. pulicaris* and *C. brunnicans* females, estimates are for treatments on the top row relative to treatments in the left column (*=p<0.05, **=p<0.01, ***=p<0.001)**

| Trap | CDC | UV | Blue | Green | Yellow | Red |
| --- | --- | --- | --- | --- | --- | --- |
| UV | 2.456*** | - |  |  |  |  |
| Blue | 1.033 | -1.423* | - |  |  |  |
| Green | 0.618 | -1.838** | -0.415 | - |  |  |
| Yellow | 2.467*** | 0.010 | 1.433* | 1.848*** | - |  |
| Red | 5.582*** | 3.125* | 4.549*** | 4.964*** | 3.115* | - |
| White | 1.191* | -1.265 | 0.157 | 0.572 | -1.276 | -4.391*** |

Total *C. dewulfi* females

| Trap | CDC | UV | Blue | Green | Yellow | Red |
| --- | --- | --- | --- | --- | --- | --- |
| UV | 1.746*** | - |  |  |  |  |
| Blue | 0.957 | -0.788 | - |  |  |  |
| Green | 0.451 | -1.295** | -0.506 | - |  |  |
| Yellow | 1.751*** | 0.005 | 0.793 | 1.300** | - |  |
| Red | 5.701*** | 3.955** | 4.743*** | 5.250*** | 3.950** | - |
| White | 1.009* | -0.736 | 0.051 | 0.558 | -0.741 | -4.691*** |

Total *C. pulicaris* females

| Trap | CDC | UV | Blue | Green | Yellow | Red |
| --- | --- | --- | --- | --- | --- | --- |
| UV | 1.669* | - |  |  |  |  |
| Blue | 1.345 | -0.324 | - |  |  |  |
| Green | 0.344 | -1.325 | -1.000 | - |  |  |
| Yellow | 1.432* | -0.237 | 0.087 | 1.087 | - |  |
| Red | 3.188*** | 1.519 | 1.843* | 2.844*** | 1.756* | - |
| White | 1.054 | -0.615 | -0.291 | 0.709 | -0.378 | -2.134** |

Total *C. brunnicans* females
